# Growing Concerns With the Flow of Misinformation From Electronic Books

**DOI:** 10.2196/ijmr.2541

**Published:** 2013-05-24

**Authors:** Kenzo Takahashi, Hideyuki Kanda, Shunsaku Mizushima

**Affiliations:** ^1^Department of Epidemiology and Public HealthGraduate School of MedicineYokohama City UniversityYokohamaJapan; ^2^Advanced Medical Research CenterYokohama City UniversityYokohamaJapan

**Keywords:** misinformation, e-book, ethical guideline, anti-vaccinists, atopy business, wellness maintenance

## Abstract

In 2012, several kinds of electronic books (e-books) became available in Japan. Since several major book retailers launched e-book businesses, it is expected that e-books will become a popular source of information in the country. However, we are concerned that e-books may also be a source of misinformation. In examining 24 available materials published by anti-vaccinists, "atopy businesses", and "wellness maintenance" authors, each was found to contain inaccuracies or misinformation. Thus far, such information is only available in printed books. If these books are scanned and circulated, or published in e-book format, this misinformation may circulate rapidly as e-book devices are becoming popular, and, consequently, harm people’s health. We think that it is important for the government to formulate ethical guidelines for the publishing e-books with due consideration to freedom of expression.

## Introduction

In 2012, several electronic book (e-book) devices became widely available in Japan [1]. Even though several e-book devices were previously available, it is expected that newly introduced e-books will become more popular because the devices are sold by some of the more popular book retailers in Japan who provide free access to retail websites. Their business model is designed to provide easier access to e-books than ever before. The only thing that a user has to do is to register a payment method. In 2016, it is expected that the number of e-book users will grow to 5 million (via e-book reader), 27 million (via tablet devices), and 80 million (via smartphone) [2]. Thus, it is expected that e-books will make access to information media more convenient.

In this regard, we are concerned that e-books may be a source of misinformation. Now that several self-publishing manuals are available, circulation of individual ideas without any scientific evidence via e-books is easy. In this article, we reviewed 3 topics of misinformation circulated by printed books that have been observed in Japan, and finally conclude that an ethical guideline for e-book publications should be considered.

We searched and examined all the printed books written in Japanese that were accessible via the Internet (in total 24 books). In this paper, we will discuss the 3 major topics of concern.

## Vaccination

The first example of concern is vaccination (8 books). Japan has long history of distrust of vaccines [3-5]. Several anti-vaccinists, some with medical licenses and others without, published books that suggest that it is inappropriate for readers to vaccinate their children. Their structure of argument usually contains both correct information and false recommendations and concluded that government recommendations are unsound. For example, some publications asserted that mumps vaccine is not recommended and natural infection is better than vaccine for acquiring immunity. They stand on the fact that low quality mumps vaccines caused severe side effects with poor prognosis including death. The current vaccine is recognized as safe. However, the aforementioned authors asserted that the data quality is not reliable. In addition, they did not alert readers to the risk of permanent hearing loss attached to mumps infection, for example. They asserted that severe consequences seldom occur since the number of reported cases is decreasing. They also claimed that even the measles vaccine is not necessary. Their point is that children can still be infected by measles even if they received the measles vaccine because immunity may diminish within several years after injection. In addition, they introduced a case of subacute sclerosing pan encephalitis that may have been caused by the measles vaccine and pointed out that the measles vaccine should not be recommended to all children because of its severe side effects. However, available epidemiological data including genotyping data do not suggest the measles vaccine virus as a possible cause of subacute sclerosing pan encephalitis [6]. The problem with this publication is that their discussions lack stochastic consideration. However, readers who do not recognize this flaw may follow these anti-vaccine recommendations, leaving their children vulnerable to vaccine targets.

## Atopy Businesses

The second example is the "atopy businesses” (8 books) [7,8]. As the term "business" implies, books containing "atopy business" information are published for commercial reasons. They try to sell alternative therapy products such as specially treated foods, specially treated creams, and hot spring waters. They would show examples of rare cases of severe atopic dermatitis in a sensationalistic manner, and then conclude that steroids are a cause of severe diseases and should not be applied to human skin. Some of these publications claimed that, if the patients were left untreated by their products or treated by steroids, they would be sure to suffer from atopic dermatitis. An alternative therapy, that is, their own products, would be recommended. To high information seekers such as parents who worry about their children with or acquiring allergies [9], these texts look impressive and trustworthy. However, these alternatives are not medically evaluated and are generally expensive. Patients following these unproven treatment regimens would suffer financially and physically, as their conditions may worsen with these new treatments.

## Wellness Maintenance Books

The third example is "wellness maintenance books"(8 books). These books are written by qualified medical doctors and demonstrate how to live a healthy life by following some extreme life habits. In one example, the author recommended that readers eat food only once a day, leaving one's body fasted. The possibility and concerns related to the consequence of hypoglycemia or hypoalbuminemia, for example, were not thoroughly discussed. In addition, they claimed that malnutrition could be averted by eating foods in their natural state, including unpeeled vegetables and unprocessed fish with the fish head or internal organs because the authors maintain that they are perfect nutritional foods in their natural form.

## Ethical Publishing Guidelines

Some of the above mentioned content is already sold in e-book format [10]. However, in cases where they are not sold in an electronic format, used books are still sold through the Internet [11]. People may take advantage of the convenience of e-books due to the availability of self-publishing manuals, scan these books, and sell them illegally (sale of scanned books is illegal in Japan). To make matters worse, the public may write and publish their own e-books with misleading content, thus facilitating the dissemination of misinformation.

As Geraldine et al observed, “written information on medicines can be interpreted by consumers in ways that may lead to anxiety or apprehension, and a refusal of prescribed medicines” [12]. Thus, the prevalence of e-books may have a detrimental impact on human health. Fortunately, at the time of writing this paper, these books are not yet published electronically. While it is an ideal time to create legislation to punish publishers/authors who caused harmful effects to people’s health, it is difficult to judge the causal relationships between published books and health effects. We recommend that the government should promptly formulate ethical guidelines targeted at the content of e-books, listing that disputable information that should not be allowed in e-books with due consideration to freedom of expression/publication. Publishing associations should be watchful of the material that they publish based on the stated ethical guidelines and control the distribution of disreputable e-books.
